# Human Mesenchymal Stem/Stromal Cells from Umbilical Cord Blood and Placenta Exhibit Similar Capacities to Promote Expansion of Hematopoietic Progenitor Cells In Vitro

**DOI:** 10.1155/2017/6061729

**Published:** 2017-11-09

**Authors:** Guadalupe R. Fajardo-Orduña, Héctor Mayani, Patricia Flores-Guzmán, Eugenia Flores-Figueroa, Erika Hernández-Estévez, Marta Castro-Manrreza, Patricia Piña-Sánchez, Lourdes Arriaga-Pizano, Alejandro Gómez-Delgado, Guadalupe Alarcón-Santos, Odette Balvanera-Ortíz, Juan J. Montesinos

**Affiliations:** ^1^Mesenchymal Stem Cells Laboratory, Oncology Research Unit, Oncology Hospital, National Medical Center, IMSS, Mexico City, Mexico; ^2^Hematopoietic Stem Cells Laboratory, Oncology Research Unit, Oncology Hospital, National Medical Center, IMSS, Mexico City, Mexico; ^3^Niche and Hematopoietic Microenvironment Laboratory, Oncology Research Unit, Oncology Hospital, National Medical Center, IMSS, Mexico City, Mexico; ^4^FES Zaragoza, National Autonomous University of Mexico, Mexico City, Mexico; ^5^Molecular Oncology Laboratory, Oncology Research Unit, Oncology Hospital, National Medical Center, IMSS, Mexico City, Mexico; ^6^Immunochemistry Research Unit, Specialties Hospital, National Medical Center “Siglo XXI”, IMSS, Mexico City, Mexico; ^7^Infectious and Parasitic Diseases, Medical Research Unit, Pediatric Hospital, National Medical Center, IMSS, Mexico City, Mexico; ^8^Troncoso General Hospital, IMSS, Mexico City, Mexico

## Abstract

Mesenchymal stem/stromal cells (MSCs) from bone marrow (BM) have been used in coculture systems as a feeder layer for promoting the expansion of hematopoietic progenitor cells (HPCs) for hematopoietic cell transplantation. Because BM has some drawbacks, umbilical cord blood (UCB) and placenta (PL) have been proposed as possible alternative sources of MSCs. However, MSCs from UCB and PL sources have not been compared to determine which of these cell populations has the best capacity of promoting hematopoietic expansion. In this study, MSCs from UCB and PL were cultured under the same conditions to compare their capacities to support the expansion of HPCs in vitro. MSCs were cocultured with CD34^+^CD38^−^Lin^−^ HPCs in the presence or absence of early acting cytokines. HPC expansion was analyzed through quantification of colony-forming cells (CFCs), long-term culture-initiating cells (LTC-ICs), and CD34^+^CD38^−^Lin^−^ cells. MSCs from UCB and PL have similar capacities to increase HPC expansion, and this capacity is similar to that presented by BM-MSCs. Here, we are the first to determine that MSCs from UCB and PL have similar capacities to promote HPC expansion; however, PL is a better alternative source because MSCs can be obtained from a higher proportion of samples.

## 1. Introduction

Mesenchymal stem/stromal cells (MSCs) are primitive cells that give rise to bone marrow (BM) stromal cells, which are responsible for supporting hematopoiesis [1, 2]. MSCs themselves also support hematopoiesis, as they form part of the niche of hematopoietic stem cells (HSCs) and provide the necessary conditions to regulate self-renewal, proliferation, and differentiation [3–6]. Previous results from our group demonstrated the capacity to support hematopoiesis of BM-MSCs in vitro because these cells favor the expansion of hematopoietic progenitor cells (HPCs) from umbilical cord blood (UCB) [7]. HPCs obtained from UCB using ex vivo expansion systems have already been used clinically in patients undergoing hematopoietic cell transplant (HCT) [8]. Moreover, BM-MSCs have been applied in patients undergoing HCT, resulting in an increase in the graft size and faster hematopoietic recovery [6, 9–11]. Therefore, BM-MSCs are considered a serious candidate for improving HCT.

The main source of MSCs is BM; however, the use of BM has some drawbacks, as obtaining BM is an invasive procedure for the donor [12], and the number of MSCs and their capacities for proliferation and differentiation decrease with the age of the individual [13, 14]. Our research group has obtained MSCs from neonatal sources, such as umbilical cord blood (UCB) and the placenta (PL). It is noteworthy that the proportion of PL samples from which we were able to obtain MSCs was higher than that of UCB samples (100% and 11%, resp.) [15]. Moreover, for the two sources, we showed that their morphologies, immunophenotypes, and capacities for osteogenic and chondrogenic differentiation are similar to those of BM-MSCs [15] and that they have immunosuppression capacities [16, 17]. Other groups have shown that MSCs from UCB [18] and PL [19] have the capacity to support hematopoiesis in vitro but have not compared these cell types to determine which type has the best capacity for potential clinical application. In this study, we used the same coculture conditions to compare the capacities of MSCs from UCB and PL to support the in vitro expansion of HPCs from an enriched population of UCB CD34^+^CD38^−^Lin^−^ cells. MSCs from BM were included as a control. Our results demonstrate that MSCs from UCB and PL have similar capacities to support HPC expansion, and this capacity is similar to that of BM-MSCs.

## 2. Materials and Methods

### 2.1. Collection and Culture of MSCs from BM, UCB, and PL

BM samples were obtained from hematologically healthy donors according to the Declaration of Helsinki and the Local Ethics Committee of Villacoapa Hospital, Mexican Institute for Social Security (IMSS). UCB and PL samples were collected according to the Declaration of Helsinki and the Local Ethics Committee of the Troncoso Hospital (IMSS, Mexico). MSCs from BM (*n* = 6), UCB (*n* = 6), and PL (*n* = 6) were obtained as we previously reported [16, 20]. Briefly, mononuclear cells (MNCs) were obtained from BM and UCB samples by density gradient centrifugation (specific gravity < 1.077 g/mL; GE Healthcare Bio-Sciences AB, Uppsala, Sweden). MNCs were seeded at a density of 0.2 × 10^6^ cells/cm^2^ in low glucose Dulbecco's modified Eagle's medium (Lg-DMEM; Gibco BRL, Rockville, MD, USA) supplemented with 10% fetal bovine serum (FBS; Gibco BRL), 4 mM l-glutamine, 100 U/mL of penicillin, 100 mg/mL of streptomycin, and 100 mg/mL of gentamicin (all reagents were obtained from Gibco BRL). Four days later, nonadherent cells were removed, and fresh medium was added. Upon reaching 80% confluence, adherent cells were detached with trypsin-EDTA (0.05% trypsin, 0.53 mM EDTA, Gibco BRL, Rockville, MD, USA) and were reseeded at a density of 2 × 10^3^ cells/cm^2^. MSCs from the second or third passage were used for the experiments. MNCs from PL were obtained by enzymatic digestion with trypsin-EDTA (Gibco BRL, Rockville, MD, USA) and were processed in the same way as those from BM and UCB.

### 2.2. Characterization of MSCs

#### 2.2.1. Immunophenotype

Immunophenotypic analysis of MSCs was performed by flow cytometry [15, 20]. Monoclonal antibodies against CD14, CD31, CD34, CD45, CD105, HLA-DR (Caltag Laboratories, USA), CD73, and CD90 (Becton Dickinson/PharMingen, USA) conjugated with FITC (fluorescein isothiocyanate), PE (phycoerythrin), or APC (allophycocyanin) were used. Cells were acquired using a FACSCalibur (Becton Dickinson), and the data were analyzed with FlowJo 7.6.1 software (FlowJo LLC, Ashland, Oregon, USA).

#### 2.2.2. Differentiation Capacity

Osteogenic and adipogenic differentiation was induced with Stem Cell Kits™ (STEMCELL Technologies Inc., Vancouver, BC, Canada), and chondrogenic differentiation was induced using chondrogenic differentiation medium (Cambrex Bio Science Walkersville Inc., Maryland, USA) supplemented with 10 ng/mL transforming growth factor beta (TGF*β*; Cambrex). Differentiation capacities were determined using immunocytochemical stains, as we previously reported [16, 20].

### 2.3. CD34^+^CD38^−^Lin^−^ Cell Enrichment

CD34^+^CD38^−^Lin^−^ cells were enriched from UCB MNCs by negative selection using a StemStep™ kit (Stem Cell Technologies Inc., Vancouver, Canada) according to the manufacturer's instructions, as we previously reported [21].

### 2.4. Coculture of MSCs-HPCs

As we previously reported [20], MSC layers at 80% confluence were incubated with 0.3 *μ*g/mL mitomycin C to inhibit cell growth. Ten thousand cells enriched in CD34^+^CD38^−^Lin^−^ cells were seeded on MSC layers in 6-well plates (Corning Inc., Costar, New York, NY, USA) in Stem Line medium (Sigma-Aldrich, St. Louis, MO, USA) with or without the early acting cytokines thrombopoietin (TPO), Flt-3 ligand (FL), stem cell factor (SCF), and interleukin-6 (IL-6) at a concentration of 10 ng/mL (Peprotech, USA). In cultures in which MSC-HPC contact was inhibited, 0.4 *μ*m Transwells (BD) were used. Cultures were taken on day 14, with a medium change on day 7.

### 2.5. Proliferation of Hematopoietic Cells

The total numbers of nucleated and viable cells from cultures and cocultures were determined with a hemocytometer using Turck's solution and trypan blue stain (Gibco), respectively [20].

### 2.6. Colony-Forming Cell (CFC) Assays

To determine the expansion of HPCs, the presence of CFCs was analyzed using methylcellulose assays (MethoCult™; STI), as we previously reported [20–22]. After 14 days of culture, CFCs were counted with the aid of an inverted microscope. CFCs were classified as follows: erythroid colonies included committed erythroid progenitor cells or CFC-Es (erythrocyte colony-forming cells) and colonies derived from erythroid progenitor cells or BFC-Es (erythrocyte burst-forming cells), whereas myeloid colonies included CFC-granulocytes (CFC-Gs), CFC-monocytes (CFC-Ms), and CFC-GMs.

### 2.7. Quantification of CD34^+^CD38^−^Lin^−^ Cells

To determine the expansion of primitive HPCs, the frequency of CD34^+^CD38^−^Lin^−^ cells was analyzed by flow cytometry as we previously reported [20]. Briefly, a total of 1 × 10^5^ MNCs were incubated with antibodies against CD34, CD38, CD14, CD16, CD19, CD41a, and CD71 conjugated with FITC, PE, or APC (Becton Dickinson). Cells were acquired using a FACSCalibur (Becton Dickinson), and the data were analyzed with FlowJo 7.6.1 software (FlowJo LLC).

### 2.8. Long-Term Culture-Initiating Cell (LTC-IC) Assays

Detection of primitive HPCs was performed using LTC-IC assays (pre-CFCs) based on the method described by Sutherland et al. and Miller et al. [23, 24], as we previously reported [7]. Briefly, after coculture with MSCs for 14 days, hematopoietic cells were cultured with the M210B4 stromal line as a feeder layer for 35 days. Subsequently, MNCs were harvested and seeded in cultures with methylcellulose for CFC quantification. A CFC/LTC-IC ratio of 8 : 1 [7, 24] was considered.

### 2.9. Statistical Analysis

The means ± SDs (standard deviations) or SEMs (standard errors of the mean) of the number of experiments conducted are reported. Student's *t*-test or one-way analysis of variance (ANOVA) and Kruskal-Wallis tests followed by Mann–Whitney *U* tests were employed using IBM SPSS Statistics 22 software. Statistical significance was considered when the *p* value was less than 0.05.

## 3. Results

### 3.1. Characterization of MSCs from BM, UCB, and PL

As we reported previously, MSCs from BM, UCB, and PL expressed marker characteristic of MSCs, such as CD105, CD73, and CD90. The expression of hematopoietic markers (CD14, CD34, and CD45) was not observed, and CD31 and HLA-DR were also absent (Supplementary Table 1 in Supplementary Material available online at https://www.hindawi.com/journals/sci/2017/6061729/sup/). Osteogenic differentiation, as detected by von Kossa staining, and chondrogenic differentiation, as detected by Alcian blue, were similar in MSCs obtained from the three sources. Furthermore, although adipogenic differentiation was evident in MSCs from BM, no cells with adipocyte morphologies were observed in MSCs from UCB and PL. However, small positive spots were detected with oil red O staining in the cytoplasm of the MSCs (Supplementary Figure 1).

### 3.2. Enrichment of the CD34^+^CD38^−^Lin^−^ Population

A mean of 136.2 ± 63.3 × 10^6^ MNCs was obtained from UCB samples (*n* = 12 with 58.6 ± 18.3 mL volume). After enrichment by negative selection, a mean of 0.7 ± 0.54 × 10^6^ MNCs (0.54 ± 0.33% recovery) was obtained. Enrichment in CD34^+^CD38^−^Lin^−^ cells corresponded to a mean of 46.9 ± 24.7%.

### 3.3. MSCs from UCB and PL Increased Proliferation of the Population Enriched in CD34^+^CD38^−^Lin^−^ Cells

**Figure 1:**
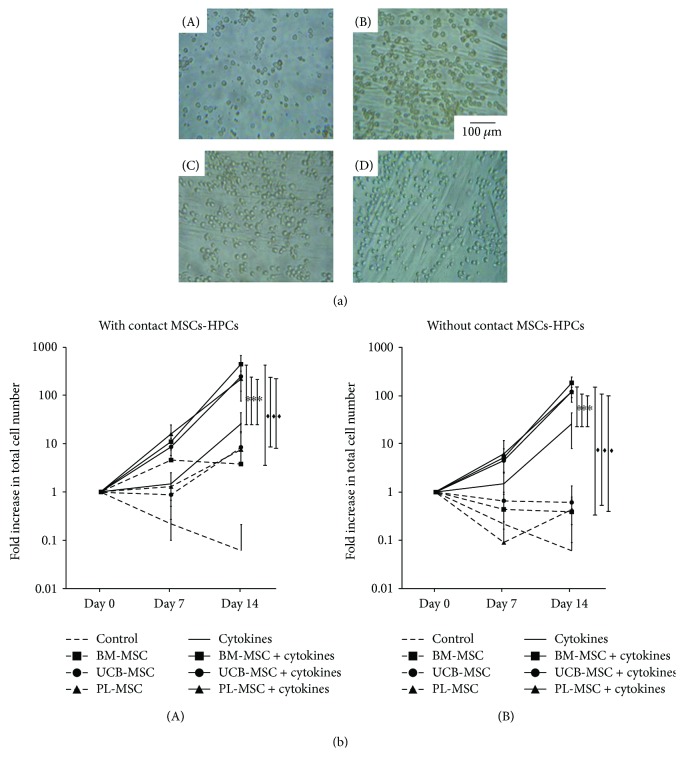
MSCs from UCB and PL increase proliferation of the population enriched in CD34^+^CD38^−^Lin^−^ cells. (a) Representative culture of CD34^+^CD38^−^Lin^−^ cells in the presence of cytokines (day 14): (A) without MSCs, (B) with BM-MSCs, (C) with UCB-MSCs, and (D) with PL-MSCs (magnification: 20x). (b) Kinetics of CD34^+^CD38^−^Lin^−^ cell proliferation in the presence of MSCs and in the absence (dotted lines) or presence (solid lines) of cytokines. Cocultures were prepared in the presence (A) or absence (B) of cell-cell contact (MSCs-HPCs). Control without MSCs (no vignette); BM-MSCs (square); UCB-MSCs (circle); and PL-MSCs (triangle). Data are shown as the means ± SD for the fold increases in cell number (BM-MSCs: *n* = 6; UCB-MSCs: *n* = 6; and PL-MSCs *n* = 6). ∗ and ♦ indicate statistically significant differences, *p* < 0.05.

We previously defined proliferation as the production of new cells from a cell population regardless of the type of cells produced [22]. Cultures of HPCs with or without MSCs from BM, UCB, and PL were analyzed (Figure 1(a): A, B, C, and D). The data are shown as the fold increases in cell number, which is defined as *B*/*A* (where the initial value is *A*, and the final value is *B*). On day 14, in cocultures with MSC-HPC contact and in the absence of cytokines, fold increases in the total number of hematopoietic cells of 3.8 ± 4, 8.4 ± 9.4, and 7.6 ± 9.2 were observed in the presence of MSCs from BM, UCB, and PL, respectively (Figure 1(b), A). Interestingly, when cytokines were added to the cocultures, significantly greater (*p* < 0.05) fold increases of 444 ± 230, 248 ± 171, and 221 ± 98 were observed in the presence of MSCs from BM, UCB, and PL, respectively, compared with cultures containing only cytokines (26 ± 18) or MSCs (Figure 1(b), A). No significant differences (*p* < 0.05) in proliferation in the presence of cytokines were detected between MSCs from the three sources.

We also analyzed the significance of MSC-HPC contact in the proliferation of hematopoietic cells by performing cocultures in the presence of a Transwell membrane to inhibit cell-cell contact. In cocultures of MSCs from the three sources on days 7 and 14, no increase in the total number of cells was observed (Figure 1(b), B). Interestingly, when cytokines were added to the cocultures, significantly greater (*p* < 0.05) fold increases of 184.25 ± 62.14, 120.29 ± 47.89, and 120.20 ± 29.55 were observed with MSCs from BM, UCB, and PL, respectively, compared with cultures only grown with cytokines (26.65 ± 18) or MSCs (Figure 1(b), B). The increase in the total number of cells was significantly greater (*p* < 0.05) in cocultures in which cell-cell contact was allowed compared with those without contact. Due to this finding, we performed HPC expansion experiments (CFC assays, quantification of CD34^+^CD38^−^Lin^−^ cells, and LTC-IC assays) only in cocultures with MSC-HPC contact.

### 3.4. MSCs from UCB and PL Increase CFC Expansion

**Figure 2:**
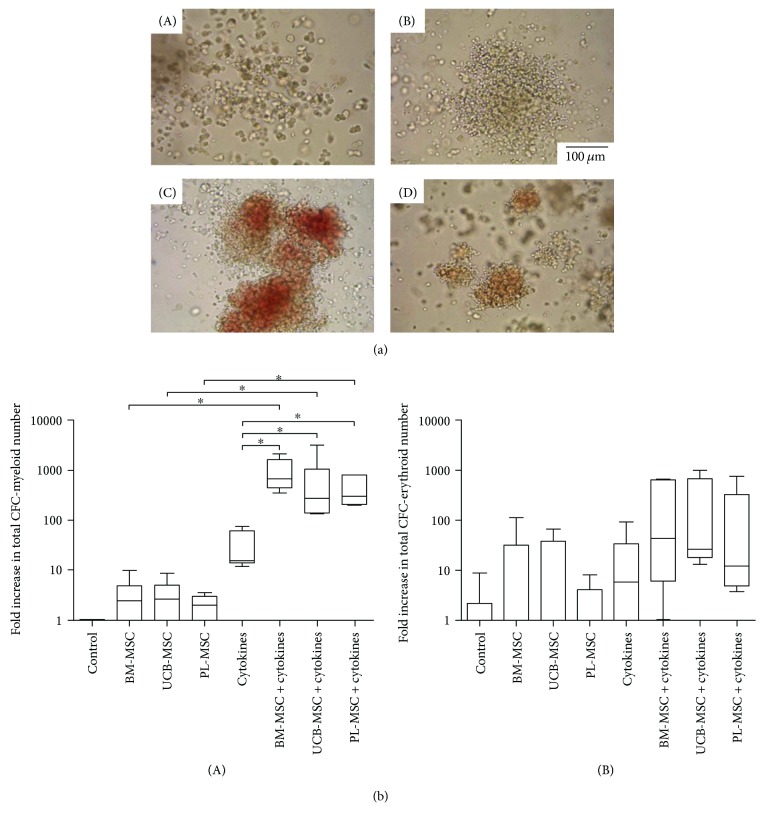
MSCs from UCB and PL increase CFC expansion of the population enriched in CD34^+^CD38^−^Lin^−^ cells. (a) Photographs of colonies obtained on day 14 of culture: (A) CFC-monocytes, (B) CFC-granulocytes, (C) BFC-erythroids, and (D) CFC-erythroids (magnification: 20x). (b) Fold increases in the number of (A) CFC-myeloids and (B) BFC-erythroids and CFC-erythroids in cocultures in the absence and presence of cytokines. Data are shown as the fold increases in total CFC number (BM-MSCs: *n* = 6; UCB-MSCs: *n* = 6; and PL-MSCs *n* = 6). ∗ indicates a statistically significant difference, *p* < 0.05.

We previously defined cellular expansion as the production of cells that maintain specific characteristics of the population of cells from which they originated [22]. Thus, hematopoietic progenitor expansion has been evaluated by the increase in the number of myeloid (CFC-G, CFC-M, and CFC-GM) and erythroid colonies (CFC-E and BFC-E) (myeloid colonies, Figure 2(a): A and B; erythroid colonies, Figure 2(a): C and D). On day 14, in cocultures without cytokines and in the presence of MSCs from BM, UCB, and PL, slight fold increases of CFC-myeloids were observed in comparison with cultures without MSCs (Figure 2(b), A). Interestingly, when cytokines were added to the cocultures, the fold increase of CFC-myeloids was significantly greater (*p* < 0.05) compared with cultures containing only cytokines or only MSCs (Figure 2(b), A). In cocultures without cytokines and with MSCs from BM and UCB, the number of CFC-erythroids tended to increase in comparison with both cultures containing PL-MSCs and controls (Figure 2(b), B). When cytokines were added to cocultures, the fold increase of CFC-erythroids tended to increase compared with that of cultures containing only cytokines or only MSCs (Figure 2(b), B). No significant differences were detected in the number of myeloid and erythroid progenitors obtained in cocultures of MSCs from the three sources.

### 3.5. MSCs from UCB and PL Increase the Expansion of CD34^+^CD38^−^Lin^−^ Cells

**Figure 3:**
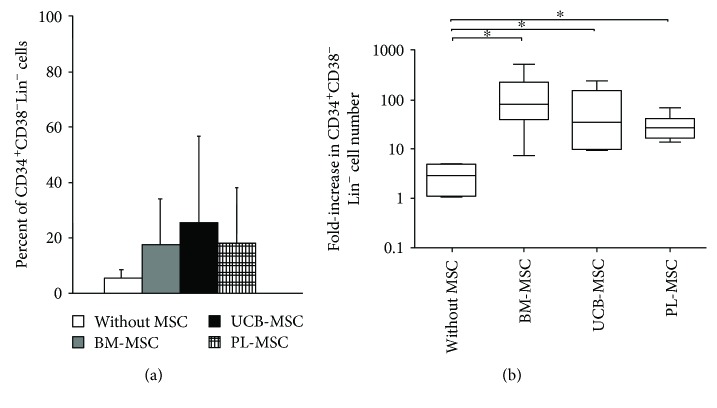
MSCs from UCB and PL increase expansion of the population enriched in CD34^+^CD38^−^Lin^−^ cells. (a) Percent of CD34^+^CD38^−^Lin^−^ cells in cocultures containing cytokines either without MSCs (white bar) or with BM-MSCs (gray bar), UCB-MSCs (black bar), and PL-MSCs (gridded bar). (b) Fold increases in the numbers of CD34^+^CD38^−^Lin^−^ cells in cocultures containing cytokines in the presence of BM-MSCs, UCB-MSCs, and PL-MSCs. Cultures without MSCs and with cytokines were considered controls (without MSCs). Data are shown as the means ± SD for the percent and fold increases in cell number (BM-MSCs: *n* = 6; UCB-MSCs: *n* = 6; and PL-MSCs *n* = 6). ∗ indicates a statistically significant difference, *p* < 0.05.

We then evaluated the increase in the percent and number of cells with the CD34^+^CD38^−^Lin^−^ immunophenotype as another parameter to determine HPC expansion. For this experiment, cultures were generated with this population in the presence of cytokines and in the absence or presence of MSCs. On day 14 of culture, the percentages of CD34^+^CD38^−^Lin^−^ cells increased by 18 ± 16%, 26 ± 30%, and 18 ± 20% in cultures with MSCs from BM, UCB, and PL, respectively, compared to those of cultures without MSCs (5.6 ± 3.3%), although these differences were not significant (Figure 3(a)). However, the fold increases in the number of CD34^+^CD38^−^Lin^−^ cells of 146.88 ± 78.48, 91.19 ± 35.73, and 31.59 ± 8 in cultures with MSCs from BM, UCB, and PL, respectively, were significantly greater (*p* < 0.05) than those of cultures without MSCs (3.50 ± 1.43; Figure 3(b)). No significant differences were detected between MSCs from the three sources. It should be noted that the percentage and number of cells with the CD34^+^CD38^−^Lin^−^ immunophenotypes were not determined in the absence of cytokines due to the low cell numbers obtained in such cultures (data not shown).

### 3.6. MSCs from UCB and PL Favor LTC-IC Formation

**Figure 4:**
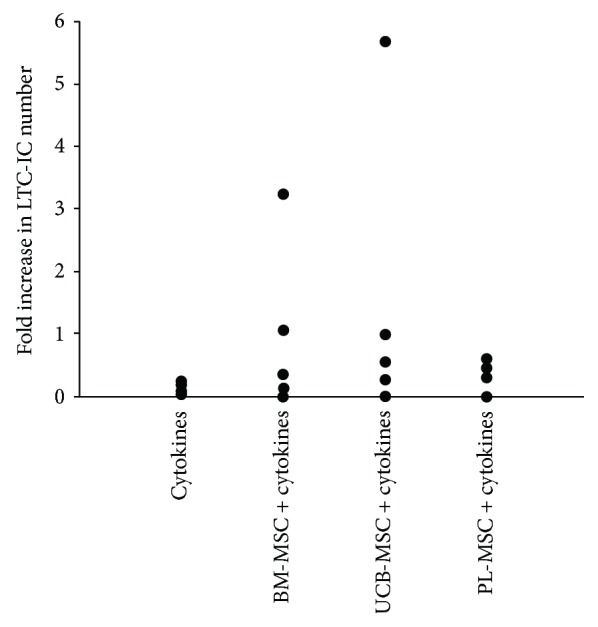
MSCs from UCB and PL favor the formation of LTC-ICs. Fold increases in the numbers of LTC-ICs on day 14 of culture. Cultures containing only cytokines were considered controls. Data are shown individually as independent experiments. Control, *n* = 4; BM-MSCs, *n* = 5; UCB-MSCs, *n* = 5; and PL-MSCs, *n* = 4.

We analyzed the effect of MSCs on the expansion of primitive HPCs with LTC-IC capacity in the presence of cytokines. The absolute values of LTC-IC obtained were 345 ± 10 on day 0 of culture; 41 ± 20 after 14 days of culture without MSCs; and 327 ± 203, 517 ± 365, and 113 ± 28 in the presence of BM-MSCs, UCB-MSCs, and PL-MSCs, respectively. On day 14 of culture, increases in the numbers of LTC-ICs were observed in some cultures containing MSCs from the three sources (Figure 4) compared to cultures with cytokines alone. The average fold increases of LTC-ICs with BM-MSCs, UCB-MSCs, and PL-MSCs were 0.95 ± 0.59, 1.5 ± 1.06, and 0.33 ± 0.08, respectively; compared with cultures containing only cytokines (0.12 ± 0.06), these values tended to be maintained (Figure 4).

## 4. Discussion

In this in vitro study, we used the same culture conditions to compare the capacities of UCB-MSCs and PL-MSCs to support hematopoiesis of a population enriched in CD34^+^CD38^−^Lin^−^ cells obtained from UCB. MSCs from the two sources met the necessary immunophenotypic, osteogenic, and chondrogenic differentiation capacities according to criteria established by the ISCT [25]. However, as we have previously reported, MSCs from these two neonatal sources do not have the same adipogenic capacities as BM-MSCs, which may be related to the tendency of MSCs to form adipocytes in adulthood [16].

Few studies have been conducted to assess the in vitro hematological support capacities of UCB and PL. Such capacities have been evaluated separately in each source on populations enriched in CD34^+^ cells [18, 19, 26, 27]. However, as we have shown previously, this hematopoietic population can be divided into CD34^+^CD38^+^Lin^−^ and CD34^+^CD38^−^Lin^−^ subpopulations, the latter of which has a greater potential for proliferation and expansion (because of their more primitive nature compared to the former subpopulation) [7, 21]. Therefore, we performed an in vitro analysis of the hematopoietic support capacity of MSCs from the two sources using the same culture conditions and a more primitive population enriched in CD34^+^CD38^−^Lin^−^ cells. Importantly, we evaluated a primitive population that showed variable purity, which could influence the number of CFCs obtained. However, because the cell populations enriched in HPCs were cultured in the presence of MSCs from the two sources, which were established at the same time under the same culture conditions, such variations did not affect the potential of MSCs to provide hematopoietic support. Thus, we sought to determine which of the two sources had the best in vitro hematopoietic support capacity in order to support their use in coculture systems as a feeder layer that promotes the expansion of HPCs, as these systems aim to obtain a sufficient number of cells to be used for hematopoietic cell transplantation.

MSCs from the two sources presented similar capacities to increase the number of hematopoietic cells under the same culture conditions. Similar results have been obtained in separate studies of UCB [18] and PL [19] in which populations enriched in CD34^+^ cells were used, thus indicating that this capacity is maintained in populations with more primitive immunophenotypes. We observed a synergistic effect in which the capacity of MSCs from the two sources improved in the presence of early acting cytokines (SCF, TPO, FL, and IL-6), as we had previously reported for BM-MSCs [7] and as reported by other groups using other cytokines, such as fibroblast growth factor 1 and IL-3, in CD34^+^ populations [18, 19, 27, 28]. In the presence of cytokines, MSCs from both sources showed similar capacities to increase proliferation. However, contrary to the effect that we observed on the population of CD34^+^CD38^−^Lin^−^ cells, other groups have found that MSCs from UCB [18] and PL [19] have higher capacities than BM-MSCs to increase CD34^+^ cell proliferation. This finding may be explained by the contributions of more mature populations in response to the effects of cytokines, which were added in higher concentrations than in our study.

Previous in vitro studies have analyzed the hematopoietic support capacities of MSCs from the two sources in cell contact cocultures [18, 19, 26, 27]; however, little is known about the significance of cell contact in that capacity. Our results demonstrate for the first time the significance of cell contact in terms of hematopoietic support of UCB-MSCs and PL-MSCs, as we observed that the increase in the number of hematopoietic cells was greater in cocultures with cell contact in both the absence and presence of cytokines. In this process, cell adhesion and extracellular matrix molecules may be involved, as N-cadherin, VCAM-I, ICAM-I, and ALCAM have been reported to be expressed by UCB-MSCs [13, 22]. Furthermore, ICAM-I, ALCAM, LFA-3, MCAM, fibronectin, and laminin have been shown to be expressed by PL-MSCs [15, 26, 29, 30]. All of these molecules are important in the adhesion, maintenance, and proliferation of primitive hematopoietic cells [6, 31–33]. Nonetheless, the observed increases in the number of hematopoietic cells in cocultures with UCB-MSCs and PL-MSCs without cell-cell contact may have been facilitated by the secretion of hematopoietic factors, such as SDF-1, IL-6, and granulocyte macrophage colony-stimulating factor (GM-CSF), by UCB-MSCs [26]. Moreover, UCB-MSCs express cytokine genes, such as TPO, SCF, FL, and macrophage colony-stimulating factor (M-CSF) [6, 18], whereas PL-MSCs produce SDF, IL-6, and SCF and express genes for FL [6, 19, 34]. Our laboratory is currently determining the expression profiles of extracellular matrix and hematopoietic molecules in our coculture system and their involvement in HPC expansion.

MSCs from the two sources showed the same in vitro capacity to increase the expansion of CFC-myeloids and CFC-erythroids, an effect that was synergistic in the presence of cytokines. Contrary to our results, it has been reported that PL-MSCs are more capable of increasing the expansion of CFCs when compared to BM-derived MSCs in the presence of cytokines [19]. This discrepancy might be explained by the different cytokines added to the cocultures and the differential response of the less primitive CD34^+^ population of HPCs to those cytokines. Similarly, we observed a synergistic effect of MSCs in the presence of cytokines that increase the expansion of cells with the CD34^+^CD38^−^Lin^−^ immunophenotype. There were no differences in the in vitro capacities of the MSCs from the two sources. Similar results have been reported for the effects of UCB-MSCs and BM-MSCs on the expansion of the primitive CD34^+^CD38^−^ population [26]; however, the same behavior was not observed with PL-MSCs compared with BM-MSCs on the CD34^+^ population. This result may have been due to the greater potential showed by PL-MSCs to promote the expansion of the population [19, 34]. This finding supports the possible differential response in the hematopoietic expansion capacity of MSCs depending on the type of HPC population analyzed, which is important to consider in the clinical application of ex vivo HPC expansion.

We also observed that MSCs from the two sources tended to maintain the number of LTC-ICs in the presence of early acting cytokines compared with those cultured in the absence of MSCs, in which a progressive loss in the number of LTC-ICs was detected. Similar results have been reported for these capacities of UCB-MSCs and BM-MSCs, but those results were obtained in the absence of cytokines [26]. However, another study found that in the presence of cytokines, PL-MSCs have a greater capacity than BM-MSCs to increase the formation of LTC-ICs [19]. We demonstrated that MSCs from the two sources had similar in vitro capacities to maintain the number of LTC-ICs under the same culture conditions.

The in vitro hematopoietic support capacity of MSCs from neonatal sources makes them attractive therapeutic agents for HCT. However, evaluation of such capacity after expansion in clinical scale cultures (CSCs) is necessary for verification of their quality for cell therapy protocols. This step is important because, as we previously reported, BM-derived MSCs have decreased differentiation capacities toward the adipogenic, osteogenic, and chondrogenic lineages and a decreased ability to inhibit T cell proliferation even though they maintain their ability to support the proliferation and expansion of HPCs [20]. We are currently testing this hypothesis.

Notably, the immunosuppressive potential of MSCs derived from UCB and PL as alternative sources to BM is crucial due to the inflammatory and immunological role of BM-MSCs within the HSC niches. In a previous report, we compared MSCs from BM, UCB, and PL in terms of their immunosuppressive properties against lymphoid cell populations enriched in CD3+ T cells. Our results demonstrated that UCB-MSCs and, to a lesser extent, PL-MSCs have in vitro immunosuppressive potential [16].

Finally, although it is important to determine the in vitro hematopoietic support potential of UCB-MSCs and PL-MSCs, it is necessary to evaluate these capacities in animal models. These experiments are being planned for future studies.

## 5. Conclusion

This study demonstrates that UCB-MSCs and PL-MSCs have similar capacities to increase the proliferation and expansion of HPCs in terms of CFC production and the proportion of CD34^+^CD38^−^Lin^−^ cells in vitro. Furthermore, MSCs from both sources showed a tendency toward the maintenance of LTC-ICs. Such capacities are similar to those presented by BM-MSCs. Additionally, for the two cell sources, cell-cell contact is important in the process of hematopoietic formation. To our knowledge, this is the first study to compare the hematopoietic support capacity of UCB-MSCs and PL-MSCs under identical culture conditions. Our results suggest that UCB-MSCs and PL-MSCs could be a good alternative to BM-MSCs in HCT. In addition, both sources could be used in ex vivo expansion protocols to increase the number of primitive HPCs from UCB for transplantation purposes. However, PL is a better alternative source than UCB because MSCs can be obtained from a higher proportion of PL samples than from UCB samples [15].

## Supplementary Material

Supplementary Figure 1. Functional characterization of MSCs from bone marrow (BM), umbilical cord blood (UCB) and placenta (PL). a-c) Microscopic appearance of MSC morphology, as observed in cultures from the indicated sources (magnification: 5X). d-l) MSCs from the three cell sources (BM, n=6; UCB, n=6; and PL, n=6) were cultured in adipogenic, osteogenic and chondrogenic induction media for 14, 21, and 28 days, respectively. d-f) Osteogenic differentiation was indicated by calcium deposits stained with von Kossa dye (magnification: 10X). g-i) Adipogenic differentiation was indicated by the accumulation of neutral lipid vacuoles stained with Oil Red O (magnification: 10X). j-l) Chondrogenic differentiation was indicated by chondrogenic matrix stained with Alcian blue in cryosections from pelleted micromass (magnification: 20X). One representative experiment is shown. Supplementary table I. Ag expression profiles by MSCs from BM, UCB and PL..

## Additional Points

*Highlights*. Under the same culture conditions, MSCs from UCB and PL exhibit similar capacities to promote the expansion of UCB-HPCs in vitro. Feeder layers of MSCs from UCB and PL could be used for ex vivo expansion of UCB-HPCs for the purpose of hematopoietic cell transplantation. PL is a better alternative source of MSCs than UCB because MSCs can be obtained from a greater proportion of PL samples. MSCs from PL may be used as an alternative source to those from bone marrow for clinical applications, such as hematopoietic recovery in patients undergoing hematopoietic stem cell transplantation.
