# Supplementary material for: Human Mesenchymal Stem/Stromal Cells from Umbilical Cord Blood and Placenta Exhibit Similar Capacities to Promote Expansion of Hematopoietic Progenitor Cells In Vitro
Source: Stem Cells Int. 2017 Nov 9;2017:6061729. doi: 10.1155/2017/6061729 (PMC5840651; doi:10.1155/2017/6061729)
Supplement: Supplementary file 1 — Supplementary Figure 1. Functional characterization of MSCs from bone marrow (BM), umbilical cord blood (UCB) and placenta (PL). a-c) Microscopic appearance of MSC morphology, as observed in cultures from the indicated sources (magnification: 5X). d-l) MSCs from the three cell sources (BM, n=6; UCB, n=6; and PL, n=6) were cultured in adipogenic, osteogenic and chondrogenic induction media for 14, 21, and 28 days, respectively. d-f) Osteogenic differentiation was indicated by calcium deposits stained with von Kossa dye (magnification: 10X). g-i) Adipogenic differentiation was indicated by the accumulation of neutral lipid vacuoles stained with Oil Red O (magnification: 10X). j-l) Chondrogenic differentiation was indicated by chondrogenic matrix stained with Alcian blue in cryosections from pelleted micromass (magnification: 20X). One representative experiment is shown. Supplementary table I. Ag expression profiles by MSCs from BM, UCB and PL.. [file 6061729.f1.pdf]

Supplementary figure 1.

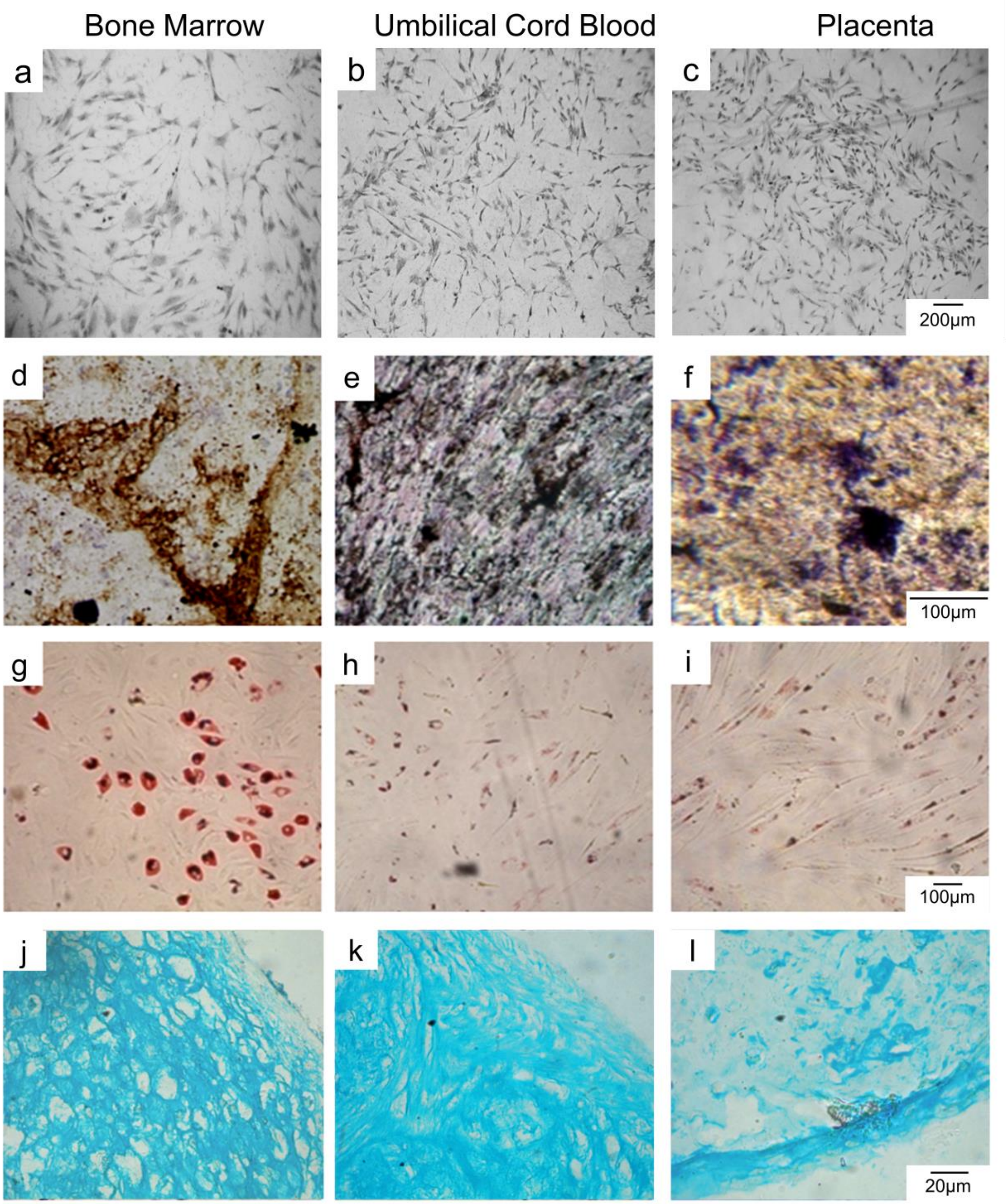

Supplementary table I. Ag expression profiles by MSCs from BM, UCB and PL

Ag	BM-MSCs	UCB-MSCs	PL-MSCs
	%	%	%
CD105	96.9±18.1	94.4±9.8	96±2.8
CD73	98±2.3	97.6±2.3	72.7±27.3
CD90	98±3.6	73.2±35	88.4±9
CD14	1.6±2.3	0.9±1.2	1±1
CD31	0.4±0.7	1±1.3	1.4±1.2
CD34	1±1.2	0.4±0.6	1.9±2.2
CD45	1.4±2.1	2.2±2.7	1.1±0.8
HLA-DR	0.3±0.3	0.9±0.7	1.3±1.5

Expression of cell markers was determined by flow cytometry. Results represent mean ± SD and correspond to the percent (%) of cells positive for each particular Ag. (BM-MSCs n=6; UCB-MSCs n=6; PL-MSCs n=6).
